# Identifying In Vitro Cultured Human Hepatocytes Markers with Machine Learning Methods Based on Single-Cell RNA-Seq Data

**DOI:** 10.3389/fbioe.2022.916309

**Published:** 2022-05-30

**Authors:** ZhanDong Li, FeiMing Huang, Lei Chen, Tao Huang, Yu-Dong Cai

**Affiliations:** ^1^ College of Biological and Food Engineering, Jilin Engineering Normal University, Changchun, China; ^2^ School of Life Sciences, Shanghai University, Shanghai, China; ^3^ College of Information Engineering, Shanghai Maritime University, Shanghai, China; ^4^ Bio-Med Big Data Center, CAS Key Laboratory of Computational Biology, Shanghai Institute of Nutrition and Health, University of Chinese Academy of Sciences, Chinese Academy of Sciences, Shanghai, China; ^5^ CAS Key Laboratory of Tissue Microenvironment and Tumor, Shanghai Institute of Nutrition and Health, University of Chinese Academy of Sciences, Chinese Academy of Sciences, Shanghai, China

**Keywords:** hepatocytes, single cell RNA sequencing, machine learning, boruta, max-relevance, min-redundancy and random forest

## Abstract

Cell transplantation is an effective method for compensating for the loss of liver function and improve patient survival. However, given that hepatocytes cultivated *in vitro* have diverse developmental processes and physiological features, obtaining hepatocytes that can properly function *in vivo* is difficult. In the present study, we present an advanced computational analysis on single-cell transcriptional profiling to resolve the heterogeneity of the hepatocyte differentiation process *in vitro* and to mine biomarkers at different periods of differentiation. We obtained a batch of compressed and effective classification features with the Boruta method and ranked them using the Max-Relevance and Min-Redundancy method. Some key genes were identified during the *in vitro* culture of hepatocytes, including *CD147*, which not only regulates terminally differentiated cells in the liver but also affects cell differentiation. *PPIA*, which encodes a CD147 ligand, also appeared in the identified gene list, and the combination of the two proteins mediated multiple biological pathways. Other genes, such as *TMSB10*, *TMEM176B*, and *CD63*, which are involved in the maturation and differentiation of hepatocytes and assist different hepatic cell types in performing their roles were also identified. Then, several classifiers were trained and evaluated to obtain optimal classifiers and optimal feature subsets, using three classification algorithms (random forest, k-nearest neighbor, and decision tree) and the incremental feature selection method. The best random forest classifier with a 0.940 Matthews correlation coefficient was constructed to distinguish different hepatic cell types. Finally, classification rules were created for quantitatively describing hepatic cell types. In summary, This study provided potential targets for cell transplantation associated liver disease treatment strategies by elucidating the process and mechanism of hepatocyte development at both qualitative and quantitative levels.

## Introduction

Over the past few decades, liver disease has gradually become one of the leading causes of death worldwide. Acute hepatitis, cirrhosis, and liver cancer account for approximately 4% of all deaths globally (Xiao et al., 2019). The only treatment for an end-stage liver disease that impairs the ability of the liver to regenerate is liver transplantation (Zhang et al., 2018). However, the practical use of liver transplantation is limited by the shortage of liver grafts for transplantation (Iansante et al., 2018). A potential alternative therapy for liver transplantation, allogeneic hepatocyte transplantation requires the cultivation of active hepatocytes *in vitro* (Iansante et al., 2018). However, obtaining hepatocytes that can function properly *in vivo* is difficult because of the different developmental processes and physiological characteristics of hepatocytes cultured *in vitro* (Hu and Li, 2015). Therefore, the development of functional hepatocytes for liver regeneration is a priority. The developmental mechanisms and heterogeneous characteristics of hepatocytes *in vitro* have become major subjects of interest because of the high clinical demand.

Liver transplant patients experience alloimmune rejection, which may cause various complications and affect the long-term survival of recipients (Du et al., 2020). Chronic allograft injury, late graft failure, and the negative effects of anti-rejection medication continue to be the major roadblocks to good outcomes (Thomson et al., 2020). Following the development of allogeneic hepatocyte transplantation technology, analysis methods for hepatic cell types and immune cell characteristics *in vitro* have become effective tools for the study of immune rejection (Kawahara et al., 1998; Iansante et al., 2018). Different hepatic cell types, including hepatoblasts, hepatocytes, and cholangiocytes, which are cultured *in vitro* and can be transplanted into a damaged liver, can repair the liver and improve liver function. The challenge of culturing functional hepatocytes *in vitro* is enormous. Primary hepatocytes have difficulty maintaining stimulation by a complex set of factors *in vivo* during *in vitro* culture, resulting in loss of hepatocyte polarity and function (Lauschke et al., 2019). In addition, owing to the shortage of donors and the lack of strategies that can increase these donors, primary hepatocytes are extremely scarce to meet the conditions for treatment. The selection of appropriate original stem cells and an *in vitro* system suitable for stem cell differentiation is crucial to the differentiation of stem cells into mature liver type cells (Guo et al., 2017). It is particularly significant to explore the process of differentiation of different original stem cells *in vitro* and to elucidate the key pathways that maintain the properties of primary hepatocytes.

Through single-cell sequencing, scientists can now investigate the mechanisms of cell growth and differentiation in unprecedented detail and resolve cell heterogeneity. Aizarani et al. successfully resolved the heterogeneity of human hepatocytes *in vivo* and the differentiation process (Aizarani et al., 2019). However, owing to environmental differences, hepatocytes cultured *in vitro* can show characteristics different from those cultured *in vivo*. Logan et al. distinguish hepatocytes cultured *in vitro* on the basis of cell shape with a machine learning approach (Logan et al., 2016). However, distinguishing hepatocytes at different stages of differentiation *in vitro* by this method remains difficult because of the diversity and ambiguity of cell morphology during development. In our study, the transcriptional profiles of different hepatic cell types cultured *in vitro* are combined using advanced machine learning methods, and the characteristic markers of various hepatocyte populations were identified. Results suggest the functional characteristics of each population. Advanced computational methods for describing liver cells cultured *in vitro* and resolving hepatocyte developmental processes and mechanisms have become a focus of research as the amount and variety of data grow.

Here, we uncovered a series of genes and classification rules linked with *in vitro* hepatocyte differentiation processes and type specificity by using advanced computational approaches based on public single-cell RNA sequencing data. First, we used two effective feature selection approaches (Boruta (Kursa and Rudnicki, 2010) and Max-Relevance and Min-Redundancy (Peng et al., 2005)) to filter and rank features. Based on ranked features, several feature sets were constructed in incremental feature selection (IFS) approaches (Liu and Setiono, 1998), which were fed into three efficient classification algorithms to build classifiers. The optimal classifier and the optimal feature subset were obtained by evaluating the performance of each classifier and observing the IFS curve. A number of genes in the optimal feature subset are associated with hepatocyte differentiation and function, demonstrating the accuracy of our computational analysis. In addition, a series of quantitative rules were established for distinguishing specific cell types and functions during hepatocyte differentiation *in vitro*. Overall, our study provided a novel computational analysis for revealing the characteristic markers of various hepatocyte populations, suggesting the functional characteristics of each cell population. The top-ranked features and decision rules identified by our analysis provided a theoretical basis for resolving hepatocyte developmental processes and mechanisms and potential targets for the treatment of clinical liver diseases.

## Materials and Methods

### Data

We obtained *in vitro* cultured human hepatocyte single-cell RNA sequencing expression profiles from the Gene Expression Omnibus (GEO) database under accession number GSE128060 (Feng S. et al., 2020). These data include 1,147 cells from 16 different hepatic cell types, each with 63,255 genes at different expression levels obtained through Smart-Seq2 sequencing. The sample sizes of each hepatic cell type are listed in Table 1. In each cell, the expression levels of genes were quantified using the transcript-per-million method.

**TABLE 1:** The sample sizes of different cell types cultured *in vitro*.

Class Index	Cell types	Sample size
1	5C-condition cultured human primary hepatocyte	96
2	Cultured human primary intrahepatic biliary epithelial cell	34
3	Definitive endoderm	15
4	Endoderm stem cell (EnSC)	24
5	EnSC-derived cholangiocyte	68
6	EnSC-derived EGFi-untreated hepatocyte	128
7	EnSC-derived hepatic endoderm	59
8	EnSC-derived hepatoblast	84
9	EnSC-derived hepatocyte	177
10	EnSC-derived immature hepatocyte	31
11	EnSC-derived TPPB-untreated cholangiocyte	75
12	Hepatocyte derived from ProliHH P2 through 3D maturation	22
13	Hepatocyte derived from ProliHH P5 through 3D maturation	32
14	Human embryonic stem cell-derived hepatocyte-like cell	140
15	Sorted ALB+ CYP3A4+ EnSC-derived hepatocyte	67
16	Uncultured adult human primary hepatocyte	95

### Boruta Feature Filtering

The majority of the features is irrelevant to the classification. When all features are selected for further analysis, redundancy and noise are introduced, which might lead to biased calculations. We used the Boruta approach to filter extraneous features in this case (Kursa and Rudnicki, 2010). The Boruta feature filtering method has been widely used in biological data mining in the past (Chen L. et al., 2021; Ding et al., 2021).

Boruta is based on the random forest (RF) classifier, which adds randomness to a system and collects results from a collection of random features. This function reduces the misleading effects of random fluctuations and correlations for the generation of the most relevant features for classification. Boruta includes the following steps: *1*) When modeling for the first time, copies of the original variables as shadow variables are generated. *2*) The values of the corresponding shadow variables are randomly shuffled. *3*) The importance score of each variable is calculated with RF modeling. *4*) For each true characteristic variable, the difference between its significance maximum and that of each shadow variable is evaluated using statistical tests. The true characteristic variables with significantly higher importance than the shadow variables are defined as significant. Real characteristic variables with significantly lower importance than the shadow variables are defined as insignificant. *5*) All insignificant variables and shadow variables are removed. The modeling and selection process is repeated and performed on the basis of the new variable composition of the dataset until all variables are classified as significant or insignificant, or a pre-set number of iterations is reached.

We used the Boruta tool from https://github.com/scikit-learn-contrib/boruta_py in this study and used the default parameters for the analysis.

### Max-Relevance and Min-Redundancy

mRMR is a filtered feature selection algorithm that maximizes the relevance between features and targets and decreases the redundancy between selected features (Peng et al., 2005; Zhu et al., 2020; Chen et al., 2022). The algorithm analyzes each feature and output category as an independent variable and measures the similarity between two variables by using mutual information, as expressed by

$$\mathrm{MI}\left(x,y\right)=\iint p\left(x,y\right)\log\frac{p\left(x,y\right)}{p\left(x\right)p\left(y\right)}\mathrm{dxdy}$$ (1)

Where p(x,y) represents the joint probabilistic density of x and y , and p(x) and p(y) represent the marginal probabilistic densities of x and y , respectively. Each time a feature is introduced to the mRMR process, the correlation between a feature set and a target must be determined. However, in feature selection, the combination of individual good features does not necessarily increase the performance of classifiers because the features may be highly correlated with each other and thus show redundancy. That is, the correlation between features and categorical variable are maximized, and the correlation between features are minimized. The formulas for maximizing correlation and minimizing redundancy are as follows:

$$\max D\left(S,c\right),D=\frac{1}{\left|S\right|}\sum_{f_{i}\in S}\mathrm{MI}\left(f_{i},c\right)$$ (2)

$$\min R\left(S\right),R=\frac{1}{{\left|S\right|}^{2}}\sum_{f_{i},f_{j}\in S}\mathrm{MI}\left(f_{i},f_{j}\right)$$ (3)

Where S is the feature subset, |S| is the number of features, $f_{i}$ is the *i*-th feature, and c is the target category. Finally, the features are selected by maximizing the equation ϕ  as follows:

maxϕ(D,R),ϕ=D−R (4)

However, it is not easy to obtain such feature subset as this problem is NP-hard. Accordingly, mRMR employs a heuristic way to complete this task. It repeatedly selects one feature with maximum relevance to target category and minimum redundancies to already-selected features. This procedure stops until all features have been selected. According to the selection order, features are sorted in a feature list. Evidently, features with high ranks are more important than those with low ranks.

We used the mRMR tool from http://home.penglab.com/proj/mRMR/ and used the default parameters for the analysis.

### Incremental Feature Selection

Through mRMR method, we can obtain a feature list. However, it is still a problem which features should be selected. To determine the optimal features for one classification algorithm, the IFS method (Liu and Setiono, 1998) was employed.

IFS is a frequently used method for determining the ideal feature number for classification when combined with a classification algorithm (Liu and Setiono, 1998; Zhang et al., 2020; Zhang et al., 2021). Based on the feature list yielded by the mRMR method, it first builds a succession of feature subsets by one-step interval. The top feature in the list is included in the first feature subset, the top two features are included in the second feature subset, and so on. On each feature subset constructed, one classifier is generated based on the given classification algorithm and samples represented by the features in the subset. Such classifier is assessed through ten-fold cross-validation (Kohavi, 1995). The best classifier can be found, which was termed as the optimal classifier. The features used in such classifier were called optimal features and they comprised the optimal feature subset.

### Synthetic Minority Oversampling Technique

As shown in Table 1, various cell types have different sample sizes. The sample size of hEnSC-derived hepatocytes was approximately 12 times that of EnSCs, and thus the sample size was highly unbalanced. This condition can lead to strong preferences in the training process, resulting in unreliable results. In the analysis of the effectiveness of each classifier, the synthetic minority oversampling technique (SMOTE) was used to lessen the impact of imbalance (Chawla et al., 2002; Ding et al., 2022; Pan et al., 2022; Zhou et al., 2022). The SMOTE implementation process consists of the following steps: *1*) randomly select one sample, say *x*, from a minority class; *2*) the k closest neighbors of *x* are obtained from all samples in the same minority class; *3*) sample $x_{i\left(nn\right)}\;$ is randomly selected from these *k* closest neighbors, and a random number $\zeta_{1}$ between 0 and 1 is generated to synthesize a new sample $x_{i1}$ with the following formula:

$$x_{i1}=x_{i}+\zeta_{1}\times \left(x_{i\left(\mathrm{nn}\right)}-x_{i}\right)$$ (5)

This new sample is put into the minority class; *4*) above steps are repeated several times until the minority class has same number of samples in the majority class. In this project, the “SMOTE” tool from Weka was used. The new samples yielded by SMOTE were only used in the IFS method.

### Classification Algorithm

Three efficient classification algorithms were used as candidates for the IFS method, which have been applied to tackle various biological and medical problems (Chen W. et al., 2021; Carlos et al., 2021; Liu et al., 2021; Li et al., 2022; Wu and Chen, 2022; Yang and Chen, 2022). They were briefly described as follows.

#### Random Forest

RF is an emerging and highly flexible machine learning algorithm that is widely used in biological data mining (Breiman, 2001). It is a typical type of ensemble classifier. The idea of an ensemble is to solve shortcomings inherent in a single model or a model with a certain set of parameters, and thus more models can be integrated, and limitations can be avoided. RFs are the products of the idea of ensemble, where many decision trees (DTs) are integrated into a forest for the prediction of a final outcome. Here, we called RF model from python’s scikit-learn package for classification. For convenience, we used default parameters to execute RF package. The number of DTs was 100.

#### k-Nearest Neighbor

KNN is the earliest collaborative filtering algorithm (Cover and Hart, 2003). The basic idea is to classify sample points that are close to one another into the same class. The KNN first determines a k-value which is used in selecting k-nearest samples in a specific point. Then, a selected distance is used in calculating the distance of the k-nearest samples to a specific point. Finally, a voting-based classification rule is used to determine the class to which the new sample belongs. We adopted the KNN model in scikit-learn for subsequent analysis. Default parameters were used, where the distance was defined as Minkowsk distance, *K* was set to one.

#### Decision Tree

DTs are machine learning algorithms with good interpretation, high training efficiency, and simple comprehension and frequently used in classification and feature selection (Safavian and Landgrebe, 1991). A DT splits in a recursive manner, resulting in a tree structure with nodes and directed edges. The classification of an instance is determined by sorting along the tree until it reaches a leaf node. In this study, we adopted DT implemented by the Scikit-learn package. It uses CART method with Gini index to expand the tree.

### Performance Evaluation

The Matthews correlation coefficient (MCC) is a well-balanced indicator that may be used when the sample size is imbalanced (Matthews, 1975). It is used in measuring the binary classification problem and is more reliable than other measurements in biological data. Gorodkin proposed a widely used formulation of MCC in multi-class classification problems (Gorodkin, 2004). Such MCC can be determined using the formula below:

$$\mathrm{MCC}=\frac{\mathrm{cov}\left(X,Y\right)}{\sqrt{\mathrm{cov}\left(X,X\right)\mathrm{cov}\left(Y,Y\right)}}=\frac{\frac{1}{K}\sum_{n=1}^{N}\sum_{k=1}^{K}\left(X_{\mathrm{nk}}-{\bar{X}}_{k}\right)\left(Y_{\mathrm{nk}}-{\bar{Y}}_{k}\right)}{\sqrt{\sum_{n=1}^{N}\sum_{k=1}^{K}{\left(X_{\mathrm{nk}}-{\bar{X}}_{k}\right)}^{2}\sum_{n=1}^{N}\sum_{k=1}^{K}{\left(Y_{\mathrm{nk}}-{\bar{Y}}_{k}\right)}^{2}}},$$ (6)

Where *X* is the binary matrix into which one-hot encoding converts the predicted class of each sample, *Y* is another binary matrix into which one-hot encoding converts the real class of each sample, and cov(X,Y) is the covariance of two matrices. The average of the k th column of matrices X and Y are represented by ${\bar{X}}_{k}$ and ${\bar{Y}}_{k}$ , respectively. The elements in the *n*-th row and *k*-th column of the matrices *X* and *Y* are referred to as $X_{nk}$ and $Y_{nk}$ , respectively. The MCC range is [−1, 1], and 1 indicates that the forecasts are identical to actual outcomes, 0 indicates that the predictions are no difference from random, and −1 indicates that the predictions are the polar opposites of the actual results.

In addition, some other widely used measurements for multi-class classification problems were also adopted in this study. They were overall accuracy (ACC) and individual accuracy on each class (cell type in this study). For the *i*-th class, its individual accuracy is defined as

$$\mathrm{AC}C_{i}=\frac{n_{i}}{N_{i}},$$ (7)

Where *N*_*i*_ stands for the number of samples in the *i*-th class and *n*_*i*_ is the number of correctly predicted samples in this class. As for ACC, it can be computed by

$$\mathrm{ACC}=\frac{\sum_{i=1}^{16}n_{i}}{\sum_{i=1}^{16}N_{i}},$$ (8)

Above measurements were provided as reference.

### Functional Enrichment Analysis

We can get the optimal features for one classification algorithm using the IFS method. Functional enrichment analysis is critical for uncovering key pathways involved with the *in vitro* culture process and for unraveling the molecular processes of biomedicine. Thus, Gene ontology (GO) and Kyoto Encyclopedia of Genes and Genomes (KEGG) pathway enrichment studies were performed using the R package ClusterProfiler (Wu et al., 2021).

## Results

**FIGURE 1:**
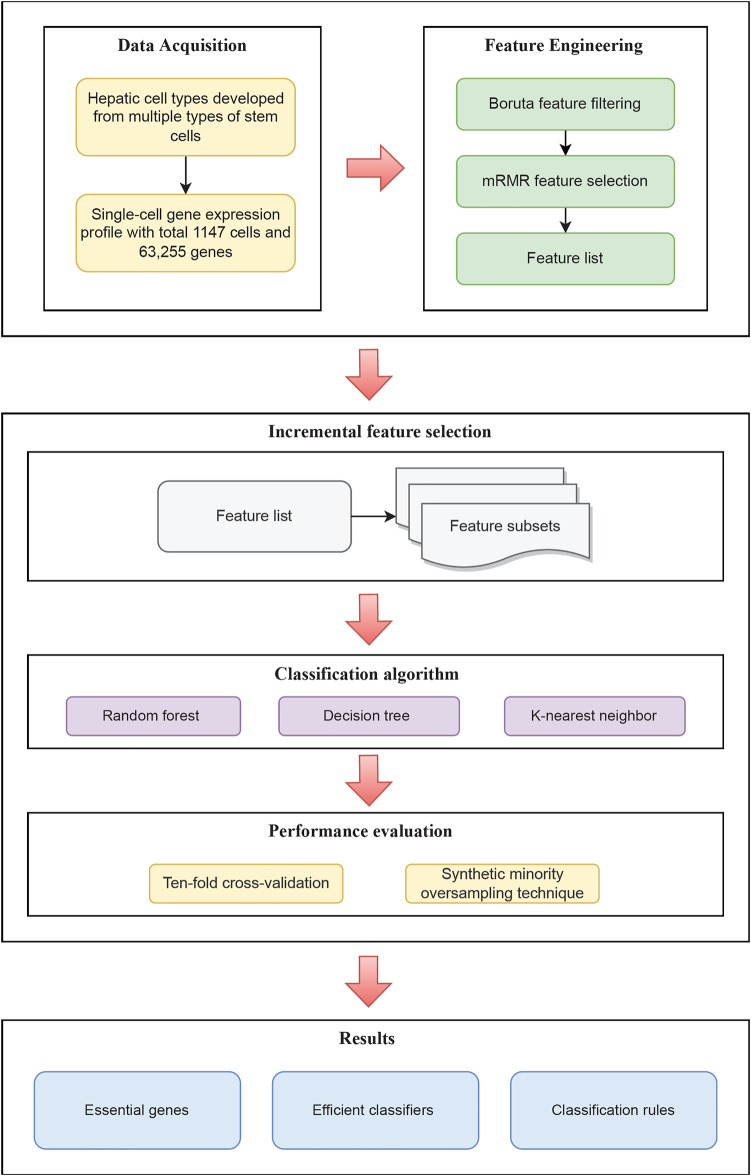
Flow chart of the entire analysis process of this study. Single-cell RNA sequencing data acquired through the GEO database includes cells from 16 different hepatic cell types cultured *in vitro*. Following that, using feature selection methods, a sorted feature list is constructed. To recover efficient genes, develop effective classifiers, and construct classification rules, this list is partitioned into feature subsets and put into the three classification algorithms.

In the current research, we explored genes that characterize the process of hepatocyte culture and differentiation *in vitro* and created a series of rules for differentiating various hepatic cell types. The entire calculation process is shown in Figure 1. The outcomes of each step were discussed in full below.

### Results of Boruta and mRMR Methods

We processed the original 63,255 features with the Boruta feature filtering approach. 1901 features were selected, which are listed in Supplementary Table S1. Subsequently, these features were analyzed by mRMR method, to obtain a list of features ranked by importance, which are also shown in Supplementary Table S1.

### Results of the IFS Method

**FIGURE 2:**
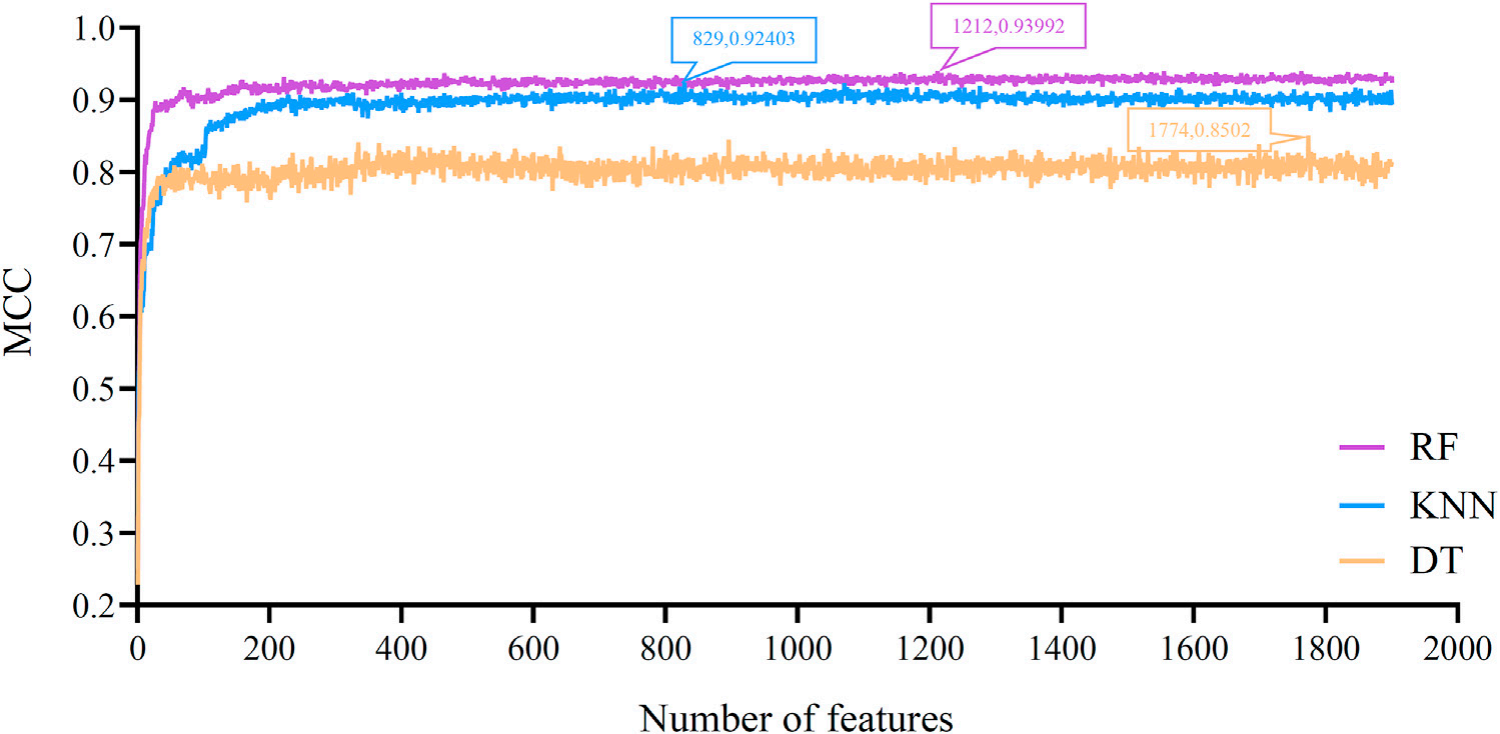
IFS curves for evaluating the performance of the three classification algorithms under different feature subsets according to MCC. RF/KNN/DT reaches the maximum MCC value of 0.940/0.924/0.850 at the feature number of 1212/829/1774.

**FIGURE 3:**
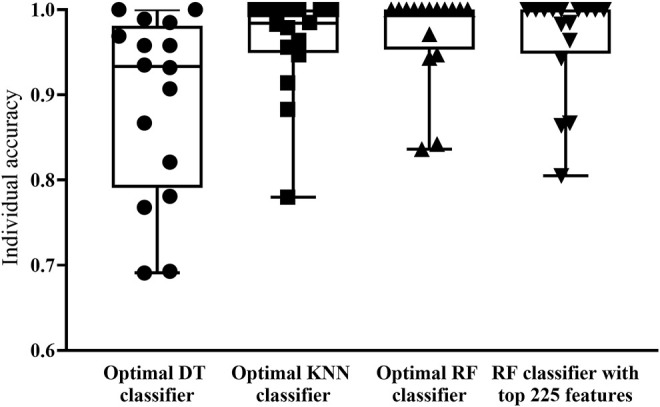
Box plot to show performance of key classifiers on 16 cell types. RF and KNN classifiers have superior classification performance, with ACC reaching above 0.950 in most cell types. DT classifier has a weaker classification performance compared to the other three classifiers.

Based on the feature list obtained in Results of Boruta and mRMR Methods section, the IFS method was performed. It constructed 1,901 feature subsets with one step interval. On each feature subset, a classifier was built by applying one classification algorithm (RF, KNN or DT) to samples represented by features in this subset. Each classifier was evaluated by 10-fold cross-validation. The evaluation results, including measurements listed in Performance Evaluation section, are provided in Supplementary Table S2. To clear display the performance of one classification algorithm under different feature subsets, an IFS curve was plotted, as shown in Figure 2, which set MCC as *Y*-axis and number of features as *X*-axis. For RF, the highest MCC was 0.940, which was obtained by using top 1212 features in the list. Accordingly, the optimal RF classifier can be built with these features. The ACC of this classifier was 0.945, as listed in Table 2. Its detailed performance on 16 cell types (i.e., individual accuracies) is shown in Figure 3. It can be observed that several cell types were perfectly predicted. All these suggested the excellent high performance of the optimal RF classifier. As for another classification algorithm, KNN, its highest MCC was 0.924, which was produced by using top 829 features. With these features, the optimal KNN classifier was set up. Such classifier yielded the ACC of 0.930 (Table 2). The MCC and ACC were all lower than those of the optimal RF classifier. Its individual accuracies on 16 cell types were also generally lower than those of the optimal RF classifier, which can be observed from Figure 3.

**TABLE 2:** 10-fold cross-validation performance of some key classifiers based on different classification algorithms.

Classification algorithm	Number of features	Overall accuracy (ACC)	Matthews correlation coefficient (MCC)
Random Forest	1212	0.945	0.940
Random Forest	222	0.937	0.931
k-Nearest Neighbor	829	0.930	0.924
Decision Tree	1774	0.863	0.850

With RF and KNN, the efficient classifiers can be built. However, they cannot provide useful clues to uncover the heterogeneity of the hepatocyte differentiation process *in vitro*. In view of this, this study further employed DT in the IFS method. The IFS curve of DT is also shown in Figure 2. When top 1,774 features were used, DT provided the highest MCC of 0.850. Likewise, the optimal DT classifier was constructed using these features. Its ACC was 0.863, as listed in Table 2. Evidently, such performance was much lower than that of the optimal RF/KNN classifier. Its performance on 16 cell types was also much lower than that of the other two optimal classifiers (Figure 3). Although the performance of the optimal DT classifier is much lower than the optimal KNN/RF classifier, it has its own merits, which would be given in Classification Rules section.

With the above arguments, we can find that the optimal RF classifier was best. Such classifier can be a useful tool to differentiate hepatic cell types cultured *in vitro*. However, the efficiency of this classifier was a problem because lots of features were used in this classifier. In view of this, we carefully checked the IFS results of RF and found that when top 222 features were adopted, RF can generate the MCC of 0.931. In this case, the ACC was 0.937 (Table 2). They were slightly lower than those of the optimal RF classifier. As for its individual accuracies, they were also a little lower than those of the optimal RF classifier, as shown in Figure 3. Furthermore, this RF classifier was superior to the optimal KNN and DT classifiers. Thus, it was more proper than the optimal RF classifier to be a tool for differentiating hepatic cell types cultured *in vitro*.

### Classification Rules

**FIGURE 4:**
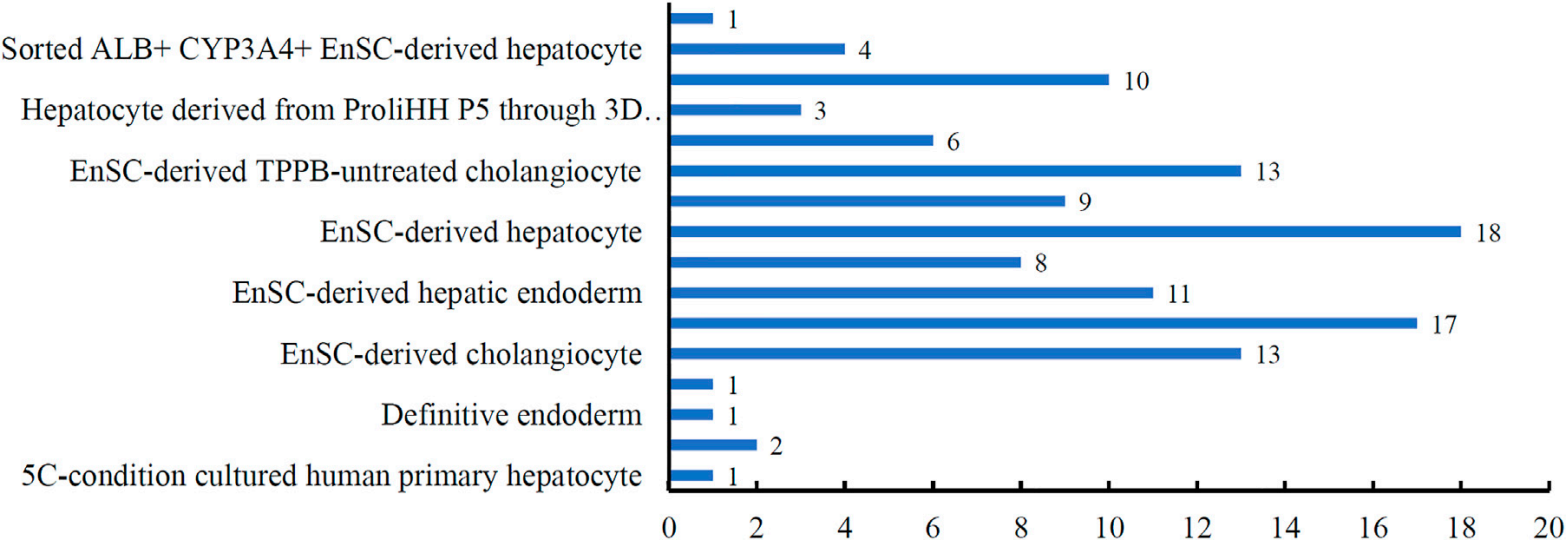
Bar chart to show the number of rules for each cell type.

By applying IFS method with DT to the *in vitro* cultured human hepatocyte single-cell RNA sequencing expression profiles, the optimal DT classifier was built. It used the top 1,774 features in the list. Although its performance was not very high, it can provide novel clues to uncover the heterogeneity of the hepatocyte differentiation process *in vitro*. With top 1,774 features, we applied DT on all cells, obtaining a large tree, from which 118 rules for classifying hepatic cell types were obtained. These rules are available in Supplementary Table S3. Each rule established a limit on the quantity of gene expression, indicating the relevance of high or low gene expression in distinguishing *in vitro* cultured cell types. Each cell type received at least one rules. Figure 4 shows the number of rules for each cell type. The cell type “EnSC-derived hepatocyte” got the most rules (18), where four cell types only got one rule. In Quantitative Rules for Stages of Liver Cells Differentiation and Specific Function Classification section, a detailed analysis of these rules would be given.

### Functional Enrichment Analyses

**FIGURE 5:**
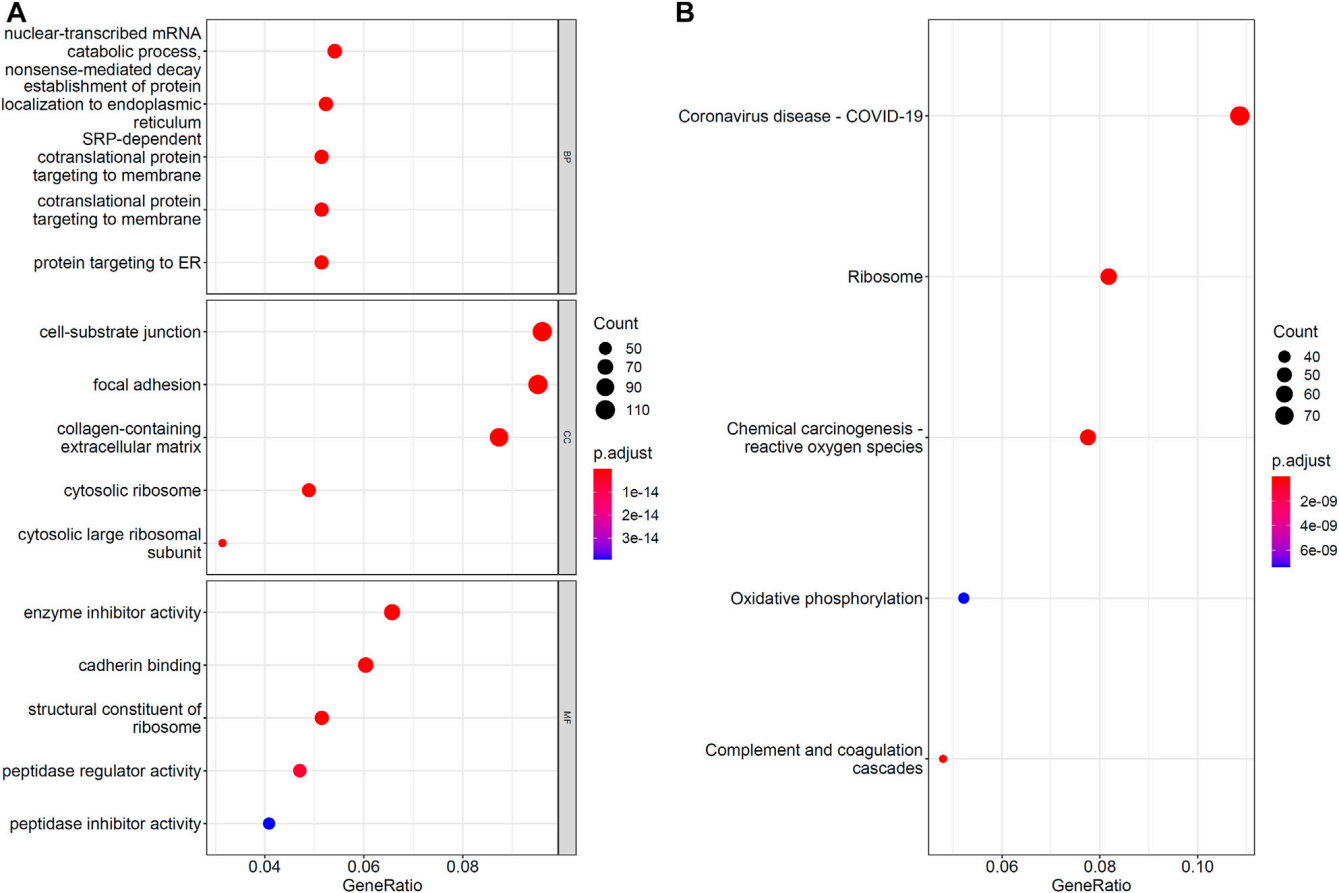
Gene ontology and KEGG pathway enrichment analysis on optimal genes for RF. The FDR<0.05 criterion was used to filter GO terms and KEGG pathways. **(A)** The top five GO enrichment results for each GO group. **(B)** The top five KEGG pathway enrichment results.

The IFS results showed that the optimal RF classifier provided the best classification performance. Such classifier used the top 1,212 features in the list, suggesting that these features greatly contributed to the model construction process for distinguishing the samples of different cell types and were directly or indirectly involved in the biological processes that distinguished these cells. To support this result, GO and KEGG pathway enrichment analysis was performed on the corresponding genes of these features by using ClusterProfiler (Wu et al., 2021) package in R. The FDR <0.05 criterion was used in filtering GO terms and KEGG pathways. Supplementary Table S4 shows the results of GO and KEGG pathway enrichment analysis results. Then, we selected the top five GO terms in each GO group and KEGG pathways for visualization, as shown in Figure 5. Some terms, such as cell–substrate junction and cadherin binding, were linked to hepatocyte differentiation *in vitro* in these enrichment results. Functional Enrichment Analysis of Optimum Genes section presented a full analysis of the enrichment results.

## Discussion

**FIGURE 6:**
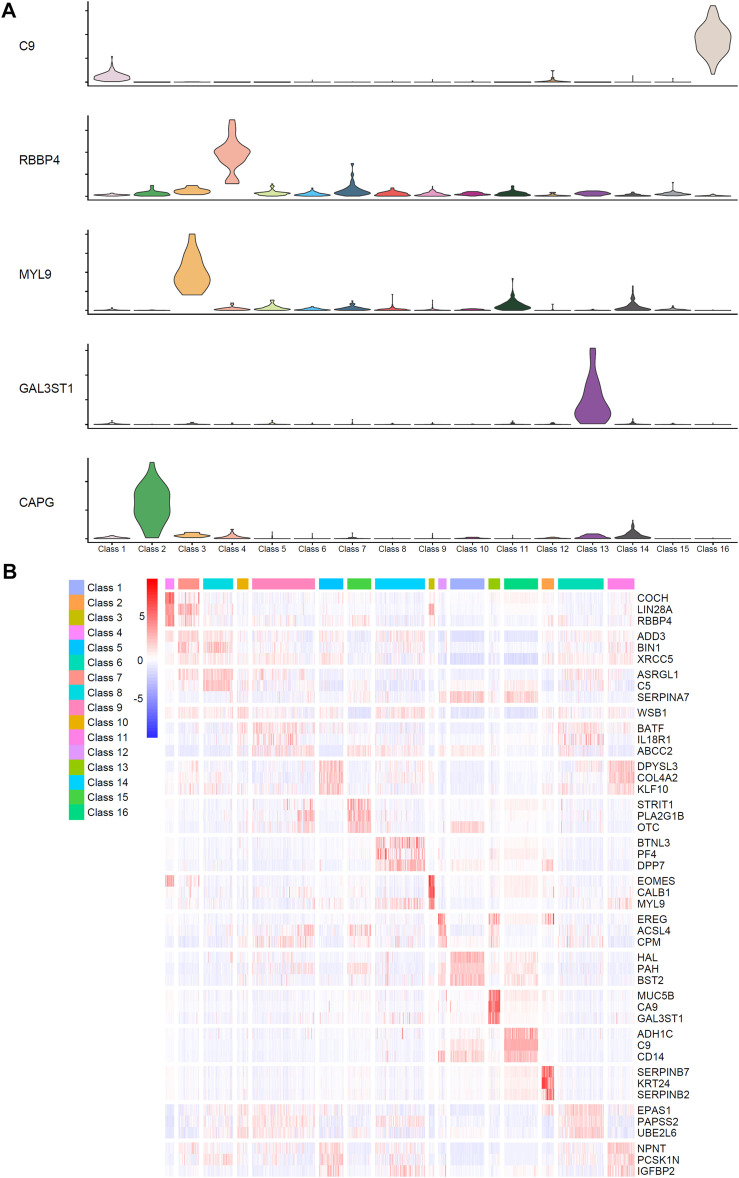
Identified expression patterns of highly ranked genes among different classes. **(A)** The violin plot of five identified genes, *C9*, *RBBP4*, *MYL9*, *GAL3ST1*, and *CAPG*, which have significant high expression level in specific classes. **(B)** The heatmap of genes ranked high in the feature list. The corresponding cell types of Class 1–16 can be found in Table 1.

We used advanced computational methods to identify qualitative features and quantitative rules for different stages of differentiation and specific functional populations of liver cells, which were cultured *in vitro*, at the single-cell level. The violin plot and heatmap were drawn using highly ranked genes to show expression patterns between different classes, which can be seen in Figure 6. These features play important roles in hepatocyte development, which also shows the accuracy of our analysis results. A detailed description of these features and rules can be seen below.

### Optimal Features for Distinguishing Different Transplantable Liver Cells *In Vitro*

By the Boruta and mRMR methods, a feature list, indicating the importance of genes, were obtained. Here, we selected five genes with high ranks in the list for detailed analysis, which are listed in Table 3.

**TABLE 3:** Important genes yielded by Boruta and mRMR methods.

Ensembl ID	Gene symbol	Description
ENSG00000034510	TMSB10	Thymosin Beta 10
ENSG00000172270	CD147/BSG	Basigin (Ok Blood Group)
ENSG00000106565	TMEM176B	Transmembrane Protein 176B
ENSG00000196262	PPIA	Peptidylprolyl Isomerase A
ENSG00000135404	CD63	CD63 Molecule

The first identified gene in the list was *TMSB10* (ENSG00000034510)*. TMSB10* encodes the conserved small acid protein belonging to the beta-thymosin family, which functions in actin function during cell motility. TMSB10 expression is related to the development of several tissues (Bani-Yaghoub et al., 2001). Back in 1990, TMSB10 was found to be highly expressed during the human fetal brain period (Hall et al., 1990). In 2011, Fanni et al. found significant differences in the expression of TSM10 among the different stages of salivary gland organogenesis (Fanni et al., 2011). TSM10 is strongly expressed in the early stages of physiological development of human salivary glands (Nemolato et al., 2009; Fanni et al., 2011). Although no studies have directly shown that TSM10 plays an important role in liver formation and development, some studies implied the important role of TSM10 in embryonic development, revealing that TSM10 may be an important regulator in the differentiation of embryonic cells into hepatocytes.

*CD147* (ENSG00000172270), also known as *basigin* (BSG), encodes a plasma membrane protein that plays important roles in life processes, such as embryo implantation and tumor progression. CD147 is one of the positive markers of a type of mesenchymal stem cells that are isolated from fetal liver (Zhao et al., 2004). This finding demonstrates the role of CD147 as a marker for identifying stem cells with high differentiation potential. It helped us select good starting cells during the *in vitro* culture of hepatocytes. CD147 regulates the production of MMP in hepatocytes and bile duct cells and reduces the degree of liver fibrosis (Calabro et al., 2014). CD147 expression affects carcinogenesis development by modulating the degree of cell differentiation in hepatocellular carcinoma (Wu et al., 2016). Through previous studies, we found that CD147 not only regulates terminally differentiated cells in the liver but also affects the differentiation process of the cells. Our method ranked it high in the list, indicating its importance in the differentiation and maturation of hepatocytes *in vitro*.

The next identified gene was *TMEM176B* (ENSG00000106565), which was first found in human lung fibroblasts (Lurton et al., 1999). TMEM176B was highly expressed in transplanted livers with recurrent hepatitis C virus, revealing its potential as a marker to distinguish abnormal reactions occurring after liver transplantation (Gehrau et al., 2011). Our study showed that TMEM176B was one of the efficient classification features, implying a specific pattern in TMEM176B expression among cell populations and further suggesting that diverse *in vitro* cultured cell populations have different adaptations for liver transplantation. In addition, TMEM176B regulates the maturation process of monocytes and dendritic cells in mice and humans (Condamine et al., 2010; Picotto et al., 2020). No direct evidence of the role of TMEM176A in hepatocyte differentiation was found, but the combination of previous and our studies revealed that TMEM176A potentially acts as a potential target for regulating hepatocyte maturation.

*PPIA* (ENSG00000196262), also known as *CYPA*, encodes a peptidyl-prolyl cis-trans isomerase that plays an important role in protein folding. It can act as a ligand to bind to CD147, thereby affecting intracellular physiological activities (Yurchenko et al., 2002). CD147, as described above, can affect the differentiation of cells within the liver. Therefore, PPIA is a potential target that influences hepatocyte differentiation. In addition, the inhibition of PPIA activity leads to the blocked polymerization of hensin in the extracellular matrix, thus preventing the full differentiation of epithelial cells (Peng et al., 2009). In 2005, CYPA was demonstrated to be involved in the early stages of neural differentiation (Urano et al., 2006). PPIA mediates many biological pathways, such as inflammation and apoptosis, but its function in the differentiation of embryonic hepatocytes *in vitro* has not been investigated. Previous studies and our studies showed its potential influence on functional cell differentiation.

The next identified gene was *CD63* (ENSG00000135404), which encodes a quadruple transmembrane protein localized on the surface of the cell membrane. This protein-mediated signal transduction event plays a role in the regulation of cell development, activation, growth, and motility (Pols and Klumperman, 2009). Exogenous TIMP-1 binds to CD63 and activates a series of pathways that ultimately mediate human hematopoietic stem or progenitor cells proliferation (Rossi et al., 2015). Thus, CD63 may act as a signaling initiator molecule that facilitates the proliferation and differentiation of stem cells *in vitro*, leading to the formation of cells with specific functions. In addition, CD63 interacts with ameloblastin in osteoblasts and promotes the interaction between CD63 and integrin β1, which ultimately promote osteogenic differentiation (Iizuka et al., 2011). CD63 is associated with cell differentiation in a variety of tissues and a potential target that influences the *in vitro* culture and differentiation of hepatocytes. Meanwhile, CD63 is one of the indicators for assessing liver regeneration and prognosis in patients with acute-on-chronic liver failure (Jiao et al., 2021). This result suggested that CD63 is critical to hepatocytes cultured *in vitro* and it may be directly related to the success of the subsequent transplantation of these cells into damaged livers.

### Functional Enrichment Analysis of Optimum Genes

The IFS curve showed that the RF reached optimal performance in 1,212 features. We performed enrichment analysis on these 1,212 feature genes and filtered. The FDR was <0.05. The GO terms and KEGG pathways were directly or briefly involved in hepatocyte differentiation and functional formation, confirming the reliability of our selection method for the classification of hepatocytes at different stages of differentiation and cells with different functions. This result confirmed the validity of our selection method for the classification of hepatocytes at different stages of differentiation and different functions. We selected some of the top GO and KEGG enrichment results for detailed analysis.

In the biological process of GO enrichment results, GO:0072599, which refers to the establishment of protein localization to the endoplasmic reticulum, displayed significant enrichment. Similar results were found for GO:0070972, which refers to protein localization to the endoplasmic reticulum. During hepatocyte differentiation, changes in endoplasmic reticulum morphology and protein content in the microsomes on the endoplasmic reticulum were observed (Dallner et al., 1966; Kanamura et al., 1990). In addition, during liver development, endoplasmic reticulum processed large amounts of proteins and lipids to temporarily direct and perform proper functions (Hetz, 2012). In the cellular component of GO enrichment results, GO:0030055, which refers to the cell–substrate junction, showed high enrichment. Hepatocytes must interact with other cells and with a chemically complex substrates to maintain activity and function (Parsons-Wingerter and Saltzman, 1993). The biomechanical effects of cell–substrate interactions affect the differentiation of embryonic liver progenitor cells (Kourouklis et al., 2016). In the molecular function of GO enrichment results, GO:0045296, which refers to cadherin binding, was found to be significantly enriched. Calnexin-mediated intercellular contacts are essential to the *in vitro* maintenance of functioning hepatocytes (Semler et al., 2005). Moreover, the incorporation of E-calcineurin in cells containing appropriate substrates can maintain cell-specific functions in the liver and induce hepatocyte differentiation processes *in vitro* (Semler et al., 2005; Haque et al., 2011). Interestingly, in the KEGG enrichment analysis, we found hsa05171, which refers to the coronavirus disease (COVID-19), to be significantly enriched. Hepatocytes and cholangiocytes cultured *in vitro* are extremely permissive to SARS-CoV-2 infection (Yang et al., 2020). Hence, COVID19-related genes may be involved in the functional formation of hepatocytes and cholangiocytes *in vitro*.

### Quantitative Rules for Stages of Liver Cells Differentiation and Specific Function Classification

In addition to qualitative features, we established a series of quantitative rules for distinguishing *in vitro* cultured liver cells. We classified these rules and cell clusters into two main categories. The first category included rules that distinguish specific cell clusters at different stages of hepatocyte differentiation *in vitro*. The second category included rules used in distinguishing specific hepatocyte clusters formed by the differentiation of different original cells *in vitro*. A detailed description of the rules can be found below.

First, the classification rules of six cell groups derived from the development of endodermal stem cells into hepatocytes and cholangiocytes were resolved. In developmental stages originating from endodermal stem cells, all the six cell types exhibited restricted SAA1, TMEM123, and CD36 expression. During the differentiation of stem cells into hepatocytes, SAA1 expression is upregulated in favor of liver metabolism, but the overexpression of SAA1 determines the development of inflammation (Shi et al., 2020; Choi et al., 2021). This finding was consistent with our results and showed the accuracy of our method. CD36 is involved in the metabolism of fat in hepatocytes, and high CD36 expression leads to fat accumulation and affects the normal functions of hepatocytes (Wilson et al., 2016; Li et al., 2019). PABPAC1 had high expression levels in Class 9 (hepatocyte) and Class 10 (immature hepatocyte) and low expression in other cells. The upregulated expression of PABPAC1 is associated with hepatocyte proliferation and growth (Hsieh et al., 2009). The classification rules for Class 4 (endoderm stem cell) and Class 7 (hepatic endoderm) showed a high degree of similarity, exhibiting the low expression of HAMP and SPTBN1 and high expression of APOE. HAMP, a protein specifically expressed in the liver, constitutes a major circulating regulator of iron uptake and distribution across tissues (Fang et al., 2020). Class 4 and Class 7 hepatocytes are cell populations in the early stages of differentiation and therefore have lower expression levels on hepatocyte-specific expressed genes. The inhibition of SPTBN1 in hepatocellular carcinoma cells increases the expression of stem cell markers, and this process is consistent with the less differentiated nature of these two types of cells (Zhi et al., 2015; Hu and Wu, 2021). APOE deficiency leads to liver senescence and is detrimental to hepatocyte differentiation (Bonomini et al., 2013). Thus, the high expression of APOE retains the strong differentiation abilities of Class 4 and Class 7 cells. In rule11, which was used in distinguishing Class 4 (endodermal stem cells), FOXH1 showed high expression levels. FOXH1 acts as a transcriptional co-activator and promotes the expression of MixL1, which plays an important role in the morphogenesis and endodermal differentiation of mouse embryos. In rule 7, which was used in distinguishing Class 5 (cholangiocyte), S100A6, GSTA1, and NOCA7 showed low expression levels, whereas QSOX1, BTG1 showed high expression levels. S100A6 plays a regulatory role in a variety of cell differentiation processes and has a low expression level in terminally differentiated cholangiocytes (Grahn et al., 2020). Given that high BTG1 expression inhibits cell proliferation and differentiation, cholangiocytes were presumed to have reached a stable state. Class 9 (hepatocyte) and Class 10 (immature hepatocyte) contained RPS27 in their classification rules, which had low expression in Class 9 and high expression in Class 10. High RPS27 expression has been reported in regenerating hepatocytes (Ganger et al., 2001). We hypothesized that RPS27 is a potential target for the transformation of immature hepatocytes into active mature hepatocytes.

The classification rules for the six classes of cell subtypes were resolved. These classes were hepatocytes obtained from the differentiation and development of three distinct original cells under different conditions. Class 1 included the primary hepatocytes maintained *in vitro* under 5C conditions, which brings the primary hepatocytes to a steady state by inhibiting a series of signaling pathways (Xiang et al., 2019). In rule 2, which was used for distinguishing Class 1, RAB5IF and CRIM1 showed low expression levels, whereas EMC7 showed high expression levels. In hepatocellular carcinoma, the RAB5I with low expression level binds to FLGR5, thereby inhibiting the proliferation of hepatocellular carcinoma cells (Koo et al., 2019). Inhibitory effect of RAB5I is similar to the inhibition of proliferation of primary hepatocytes under 5C conditions, indicating the accuracy of our method. CRIM1 is an important regulator of organ development and is highly expressed during differentiation (Iyer et al., 2016). primary cells maintained under 5C conditions are more stable and have lower differentiation indexes that those that are not, and CRIM1 has low expression level (Xiang et al., 2019). The function of EMC7 is currently undefined, but it is a potential target for maintaining the stability of primary hepatocytes *in vitro*. As for Class 16 (rule 3, uncultured adult human primary hepatocyte), SAA1 showed a high expression level in the classification rule. SAA1 encodes an acute phase protein that is highly expressed during tissue injury, inflammation, or infection (Li and Liao, 1999). Uncultured primary hepatocytes cannot maintain function *in vitro* for long periods of time. The cells may internally generate responses related to SAA1 function. In rule 16, which was used for distinguishing Class 13, XIST and CAT showed high expression levels. Highly expressed XIST binds miRNAs that inhibit cell differentiation, thereby promoting cell differentiation (Feng Y. et al., 2020). CAT is more highly expressed in immature cells than in mature cells, indicating that it is a maturation-associated gene (Tomisato et al., 2002). This finding is consistent with the characteristics of ProliHHs, which exhibits progenitor cell properties after multiple generations of culture (Zhang et al., 2018). As for Class 14 (rule 17, Human embryonic stem cell-derived hepatocyte-like cell), NRAGE and SPTBN1 showed high expression levels in the classification rule. The high expression of NRAGE facilitates the repair of homologous recombination and can make cells radioresistant by altering subcellular localization (Xue et al., 2010; Chang et al., 2018; Liu et al., 2020). The high expression of SPTBN1 can suppress inflammation in the liver (Lin et al., 2021). Our rule demonstrated the specificity of the function of hepatocytes differentiated from different original cells, proving the superiority of our method.

## Conclusion

We used innovative and widely used computational approaches on single-cell RNA sequencing data to reveal the markers of various hepatic cell types. The results suggested the functional characteristics of each population of cells. The following three major aspects of our work are the end results of our efforts. The first is a list of genes that are potential targets for hepatocyte populations cultivated *in vitro* and related to specific markers. Some markers such as *CD147*, *PPIA*, *TMSB10*, *TMEM176B*, and *CD63* were identified, and these markers have been proven to be associated with hepatocyte differentiation and maturation *in vitro*. This aspect provides a theoretical foundation for understanding hepatocyte developmental processes and mechanisms and possible targets for clinical liver disease treatment. The second is the efficient classifier for determining the types of cells in the liver. The best random forest classifier with a 0.940 Matthews correlation coefficient had been constructed to distinguish different hepatic cell types. This classifier was trained on a vast amount of single-cell data and achieved outstanding classification results. The third aspect encompassed a set of classification rules as direct indicators of distinct cell types. The classification rules reveal the features of hepatic cell types at the level of quantitative gene expression, providing a theoretical foundation for the modification of hepatocytes to better function *in vivo*.

## Data Availability

Publicly available datasets were analyzed in this study. This data can be found here: https://www.ncbi.nlm.nih.gov/geo/query/acc.cgi?acc=GSE128060.
